# Evaluation and implementation of highly challenging balance training in clinical practice for people with Parkinson’s disease: protocol for the HiBalance effectiveness-implementation trial

**DOI:** 10.1186/s12883-017-0809-2

**Published:** 2017-02-07

**Authors:** Breiffni Leavy, Lydia Kwak, Maria Hagströmer, Erika Franzén

**Affiliations:** 1 Department of Neurobiology, Care Sciences and Society, Division of Physiotherapy, Karolinska Institute, Huddinge, Sweden; 2 Stockholms Sjukhem Foundation, Stockholm, Sweden; 3 Institute of Environmental Medicine, Intervention and implementation research, Karolinska Institute, Solna, Sweden; 4 Function Area Occupational therapy & Physiotherapy, Allied Health Professionals Function, Karolinska University Hospital, Stockholm, Sweden; 5 Allied Health Professionals Function, Karolinska University Hospital, Stockholm, Sweden

**Keywords:** Balance training, Parkinson’s disease, Effectiveness-implementation, Pragmatic study design

## Abstract

**Background:**

If people with progressive neurological diseases are to avail of evidence-based rehabilitation, programs found effective in randomized controlled trials (RCT’s) must firstly be adapted and tested in clinical effectiveness studies as a means of strengthening their evidence base. This paper describes the protocol for an effectiveness-implementation trial that will assess the clinical effectiveness of a highly challenging balance training program (the HiBalance program) for people with mild-moderate Parkinson’s disease (PD) while simultaneously collecting data concerning the way in which the program is implemented. The HiBalance program is systemically designed to target balance impairments in PD and has been shown effective at improving balance control and gait in a previous RCT. Study aims are to i) determine the effectiveness of the adapted HiBalance program on performance and self-rated outcomes such as balance control, gait and physical activity level ii) conduct a process evaluation of program implementation at the various clinics iii) determine barriers and facilitators to program implementation in these settings.

**Methods:**

This effectiveness-implementation type 1 hybrid study will use a non-randomized controlled design with consecutive inclusion of people with PD at multiple clinical sites. A mixed method approach will be used to collect clinical effectiveness data and process evaluation data which is both quantitative and qualitative in nature. The consolidated framework for implementation research (CFIR) will be used to guide the planning and collection of data concerning implementation barriers and facilitators. The HiBalance program will be provided by physical therapists as a part of standard rehabilitation care at the clinical sites, while the evaluation of the implementation process will be performed by the research group and funded by research grants.

**Discussion:**

An effectiveness-implementation study design benefits patients by speeding up the process of translating findings from research settings to routine health care. Findings from this study will also be highly relevant for those working with neurological rehabilitation when faced with decisions concerning the translation of training programs from efficacy studies to everyday clinical practice.

**Trial registration:**

ClinicalTrials.gov march 2016, NCT02727478.

**Electronic supplementary material:**

The online version of this article (doi:10.1186/s12883-017-0809-2) contains supplementary material, which is available to authorized users.

## Background

People with Parkinson’s disease (PD) experience impairments in balance and gait function, symptoms which can have far-reaching negative effects on their health and quality of life [1–3]. Injurious falls and fear of falling are especially prevalent among those with PD [4, 5], a factor which may partly explain why this group are less physically active than older people of a similar age without the diagnosis [6–8]. Impairments in balance control often present in the early stage of the disease and gradually deteriorate in line with disease progression. Additionally, whereas dopaminergic drugs, the primary treatment method for PD-related motor symptoms, positively affect symptoms such as bradykinesia and tremor, these drugs are also reported to negatively affect components of balance control [9]. Both these factors combined highlight the urgency to develop and implement effective training methods to tackle balance and gait impairments for this patient group.

There is a growing evidence base for the feasibility and effectiveness of balance training in PD with regards to various aspects of physical function [10–12]. However, previous interventions have been criticized for applying training stimuli which lacked intensity and challenge [11, 13]. Group-based programs also appear to offer added motivational advantages for people with PD [12, 14], many of whom experience non-motor symptoms such as apathy and depression as a feature of the disease [15]. The HiBalance program [16] was developed with these considerations in mind to specifically address PD-specific balance impairments. This group-based program is highly challenging, progressive and has been proven effective in improving balance and gait impairments in a randomized controlled hospital setting [17]. For an intervention to be considered evidence-based however, it must be proven effective in both research and clinical settings [18].

### From randomized trial to routine care

It is recognized that efficacious interventions frequently require adaptation prior to adoption in clinical settings [19]. This adaptation to clinical factors is often required to ensure feasibility of the intervention the clinical context [20]. Best practice then involves evaluating the effectiveness of the adapted program, in order to verify whether or not the adaptation has attenuated the effective core elements of the program itself [21]. The majority of research trials however do not reach this phase as studies among American and European populations suggest that 30 − 40% of patients do not receive care which is in line with the current best scientific evidence [21, 22]. Similarly, in the field of neurological rehabilitation for PD, although there are a growing number of programs tested in randomized controlled environments, the evidence is lacking as to whether the effects of these program can be maintained in real life clinical settings.

Clinical effectiveness research is characterised by the inclusion of more heterogeneous patient samples and settings which, in turn, places greater emphasis on external validity and generalizability of the intervention in question [23, 24]. In this way, effectiveness studies can be considered essential links between scientific evidence and evidence-based practice. There is also empirical evidence to show that the process of implementation is an important determinant of program outcomes [19]. For this reason, evaluating the implementation process provides information regarding the feasibility of intervention programs within real life settings while also providing important contextual information which may help explain the success, or lack thereof, of the tested intervention. When the aim is to maximise the uptake of interventions in primary care, researchers are also advised to examine the influence of contextual factors on implementation, as this will give an indication of the ‘fit’ between the intervention and the context within which it is being embedded [25].

### Rationale for trial design

The effectiveness-implementation hybrid design [26] enables the simultaneous evaluation of both effectiveness outcome measures as well as of the process of implementation. This design is therefore hypothesized to speed up the implementation process of interventions within healthcare [26]. The type 1 Hybrid design is suitable when a study aims to both test effectiveness of a clinical intervention (outcome evaluation) while simultaneously gathering contextual data regarding potential barriers and facilitators to clinical implementation. Use of the hybrid design allows for constant monitoring of the process by which the intervention is applied, and therefore allow problems in early application to be identified and quickly altered so as to ensure better outcomes [19]. Non-randomization in the current study is a design trade-off which was made in order to ensure that implementation of the program is practically feasible at the various clinical sites within a reasonable time-frame. Lastly, we will adhere to a participatory approach whereby ‘users’ of the program (physical therapist trainers) will be actively involved in all stages of the program adaptation, process and outcome evaluation. This approach is recommended in order to increase the relevance, acceptability and successful implementation of the program [21].

### Study aims

This study has three main aims which involve testing the effectiveness of the HiBalance program:

- **Aim I:** to test the effectiveness of the adapted balance training program on balance, gait and physical activity in people with PD in real-life clinical settings.
- **Aim II:** to conduct a process evaluation of the effectiveness-implementation study by gathering information on the implementation process at the various clinical settings.
- **Aim III:** to determine barriers and facilitators that affect implementation of the program in these settings.

The study results will test the hypothesis that the effectiveness of the HiBalance program can be retained following adaptation of the intervention and study results should inform future translation of evidence-based training programs in clinical settings among people with PD.

## Methods

### Description of the HiBalance program

The intervention involves a 10-week highly challenging and progressive balance training program in group format and has been previously described [16]. The group-training exercises are not fixed but selected to target four major components of balance control which are known to be impaired among people with PD; (i) Sensory integration; (ii) Anticipatory postural adjustments; (iii) Motor agility and (iv) Stability limits. Trainers are responsible for the planning, selection and adaption of the exercises as well as ensuring that exercises are adapted to participants’ individual capacity. Successive progression of exercise difficulty is achieved by dividing the 10-week period into three blocks A (2 weeks), B (4 weeks) and C (4 weeks). During block A, the focus involves learning the exercises and ensuring quality of the performance. During block B, the level of difficulty of the exercises increases and cognitive as well as motoric dual task exercises are introduced, 1 week at a time. During block C, exercises from all four balance components are combined in order to increase the complexity of training and participants are required to switch between cognitive and motoric dual task exercises during the same training session.

### Adaptation of the HiBalance program to clinical settings

Adaptation of the HiBalance program was a measure taken in the preparation phase to ensure its applicability in the clinical context. An overview of these adaptations is outlined in Table 1. The adaptation process occurred during a series of peer-group meetings between the research team, clinical trainers who had previously participated in the RCT as well as future clinical trainers. Reducing the dose of the group training sessions from 30 to 20 hours was motivated primarily by current regulations for rehabilitation funding in the Swedish national healthcare system. Development and adaptation of the home training program was based on trainer and participant perceptions of the exercises, gathered during the pilot study using questionnaires (participants) and focus group interviews (trainers). Lab-based and time consuming outcomes assessments employed during the efficacy trial were substituted with more clinically feasible assessments. A pilot study (data not published) was then performed at two clinic settings in order to test the feasibility of these adaptations. The objective of the pilot study was to test the main uncertainties identified in the development work prior to the effectiveness-implementation study [21].

**Table 1:** HiBalance program design differences between the efficacy and effectiveness-implementation stages

Program feature	HiBalance-RCT (Efficacy phase)	HiBalance-clinical setting (Effectiveness/Implementation phase)
Inclusion criteria	Idiopathic Parkinson’s disease	Idiopathic Parkinson’s disease
	Hoehn & Yahr score of 2 or 3	Hoehn & Yahr score of 2 or 3
	Able to walk independently indoors without an aid	Able to walk independently indoors without an aid
	Mini-Mental State examination score > 24 points	Cognitively capable of following instructions in a group setting
	Age ≥60 years	All Ages
Core components	Individually adapted, highly challenging and progressive balance training in 3 blocks with progressively integrated dual-task training	Individually adapted, highly challenging and progressive balance training in 3 blocks with progressively integrated dual-task training
Dose	30 h of group training (3 x 1 h sessions/week x 10 weeks)	20 h of group training (2 x 1 h sessions/week x 10 weeks) 10 h home exercise program (1 h/week x 10 weeks)
Providers	Physical therapist PhD students (site responsible) and clinicians	Physical therapist clinicians
Sites	2 sites, one university hospital	4–6 clinical sites/primary care clinics
Outcome evaluation		
*Performance-based*		
Balance performance	Mini-BESTest score	Mini-BESTest score
	Modified figure of eight test	
Physical activity level	Steps per day measured by accelerometer	Steps per day measured by accelerometer
*Self-reported*		
Fear of falling/balance confidence	Falls Efficacy Scale-International (FES-I) (A measure of concerns about falling)	Activities-specific balance confidence scale (ABC scale) (A measure of balance confidence)
Activities of daily living	Unified Parkinson’s Disease Rating Scale (UPDRS)- ADL component	
Self-rated health Walking Evaluation method	SF-36/PDQ-39 PDQ-39 Randomized controlled trial	EQ-5D-3 L Walking impact scale (Walk 12G) Non-randomized controlled design

All physical therapist trainers will participate in 2 x 3 h sessions where they will receive theoretical and practical training in the HiBalance program as well as information regarding the project design and goals. Project materials will be provided covering all aspects of recruitment, training and measurement and regular support will be achieved with the research team though contact by mail, telephone and meetings.

The following section outlines the study setting, and for each study aim, participants, data collection procedures, and analysis plans are described.

### Study setting

The study will be conducted in 4–6 rehabilitation clinics ranging in nature from primary care geriatric clinics to out-patient clinics specializing in neurological physiotherapy rehabilitation in Stockholm, Sweden. In Sweden patients can receive out-patient physiotherapy treatment without doctor/specialist referral and number of treatments is decided upon on an individual basis. Health care treatment cost is tax-funded and clinics receive funding reimbursements for an average of eight treatment sessions/patient during 1 year. Cost to the patient is also regulated whereby in a 1 year period, individual sessions cost an equivalent of 23 US dollars up to ceiling of approx. 120 dollars, from which point onwards rehabilitation treatment is free of charge during the remaining year.

### Study phases

**Fig. 1:**
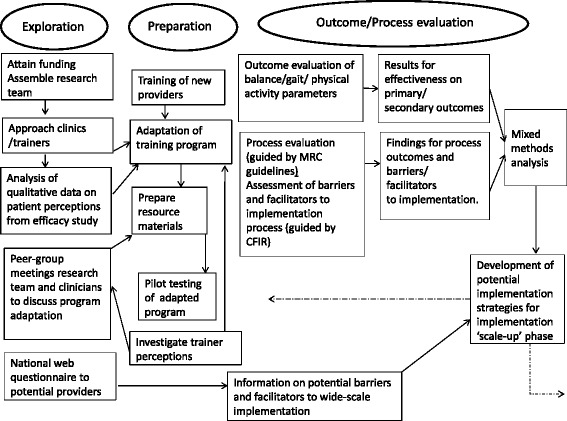
Overview of study phases for the HiBalance effectiveness-implementation trial

When describing the varying phases of this study (Fig. 1) we use taxonomy from Aaron’s conceptual model for the dynamic adaptation process [27]. According to this model, the implementation process can be divided into four phases; exploration; adoption/preparation; implementation and sustainment. In Fig. 1 we describe the first three of these stages of Aaron’s model and label the third or ‘implementation’ stage as the ‘Outcome/Process Evaluation’ as it applies to the current study. We deem that the current study will be followed by post evaluation scale-up where implementation will be tested on a wider scale, before the sustainment stage of the HiBalance program is actual. In the preparation phase for this study (Fig. 1) we will also, using a web-questionnaire, assess the needs and resources of registered physical therapists in Sweden in relation to their work with people with PD. The web questionnaire will be sent to all physical therapists registered in the neurology, geriatrics and out-patient care sections of the national organization in Sweden. This information will then be used at the later stage to inform wider-scale implementation.

### Outcome evaluation of the intervention (Aim I)

The primary and secondary outcome measures of the intervention will be assessed using a non-randomized controlled clinical trial design. Eligible participants will be consecutively included to the training groups, which mirrors the standard approach in physical therapy rehabilitation, and participants will be recruited both internally from within the clinics and in response to advertisement in local newspapers.

#### Study participants

Inclusion criteria for the trial are outlined in Table 1. A broadening of the age criteria in the current trial will enable community-dwelling people at mild-moderate stages of PD of all ages to be eligible for inclusion. Power analyses will determine sample-size using data from the Pilot study performed in the fall of 2015 as well as the previous HiBalance RCT. Control participants will be recruited and tested in a similar manner as those involved in the intervention group. Control subjects will be encouraged to continue their usual daily activities during the 10-week period between measurements and will not be advised against participating in other training interventions.

#### Data collection

The testing procedure is also designed to reflect that which occurs in real-life clinical practice and participants will be tested by physical therapists at baseline (pre-training) and at 10-weeks (post training). Data collection will occur at the respective clinics and be comprised of both clinical performance tests as well at self-reported questionnaires. All participants will be tested during the on-phase of their medication and testing will be scheduled to occur at the same time on both test occasions.

#### Outcome measures

Both performance-based and self-reported outcome measures will assess the effectiveness of the intervention (Table 1). The primary outcome measure is balance performance which will be assessed using the 14-item Mini Balance Evaluation Systems test (Mini-BESTest) [28]. This test assesses four components of balance control; anticipatory postural adjustments, postural responses, sensory orientation, and stability in gait which are directly targeted in the HiBalance program. Secondary outcomes will include gait velocity (measured by the 10-meter Walking Test); functional mobility (measured by Timed Up and Go test [29]); physical activity level (measured as steps per day using a waist-worn accelerometer (Actigraph GT3X+, Pensacola, FL, USA). In addition to performance-based outcome measures, patient-reported outcomes will be used. Balance confidence will be measured using the Swedish version of the Activities-specific balance confidence (ABC) scale [30]. This scale requires respondents to rate their confidence to maintain balance during 16 different real-life situations. The ABC scale substitutes the FES (I) scale used in previous evaluations of HiBalance [17] as this scale poses questions concerning a wider range of potentially difficult and outdoor activities and is considered more suitable to detect changes in moderate to highly functioning adults [31]. Self-rated health will be measured using the EurQol’s EQ- 5D [32]. The EQ-5D-3 L is a standardized non-disease-specific instrument for describing health-related quality of life, and is a measure already in use within rehabilitation in Sweden. When using this instrument, respondents are required to rate their health status in relation to 5 dimensions; mobility, self-care, usual activities, pain/discomfort and anxiety/depression. Subjective walking ability will be assessed using the Swedish version of the Walking impact scale (walk 12G) [33], a 12-item generic patient-reported rating scale measuring walking difficulty in everyday life as experienced during the previous 2 weeks.

#### Data analysis

Sample size calculation was based on the results from the pilot study (unpublished data) and calculated in relation to the primary outcome of balance control. In order to attain 80% power with a two-sided alpha level of 5%, the number of subjects required per group and the hypothesized effect size (of 1 point according to the MiniBESTest) was 37. With an expected drop out rate of 15% during the course of the trial, this calculation will require the inclusion of approximately 45 participants in each group. The distribution of variables which can be thought to confound study outcomes will be compared at baseline between the control and intervention groups using the Student *t* test, Mann-Whitney or Chi-squared test, where appropriate. Where differences between groups are found we plan to adjust for covariates which are known to affect outcomes in PD. Where data is seen to be of skewed distribution, logarithmic transformations or non-parametric statistics will be used to assess the effectiveness of the intervention. Repeated measures analysis of variance test or mixed models, where appropriate, will test for interaction effects between the intervention and control groups, post-hoc tests will be used when significant interactions are found. All analysis will be performed using the intention-to-treat principle and 95% confidence intervals will be calculated throughout.

### Process evaluation of the implementation (Aim II)

In order to make an accurate interpretation of the outcomes outlined above it is essential to understand the quality and quantity of the intervention that was actually delivered. For this reason, we will conduct a process evaluation of the implementation of the effectiveness study.

#### Research question relating to aim II

To what extent was the HiBalance program delivered as intended and how was the delivery conducted?

The process evaluation will be guided by the UK medical research council’s recommendations for planning and conducting process evaluation of complex interventions [21]. Process evaluations involve critically observing the work of the clinicians who are providing the training intervention [21]. Participants involved in this part of the study will therefore also include physical therapist trainers at the respective clinical sites. Due to the small numbers in individual clinics we will lack the power to statistically assess site-based factors that influence implementation outcomes. We will therefore use a mixed methods approach when evaluating the implementation process. This will be performed by data collection from trainers, patients and professionals in the field using interviews, questionnaires, analysis of patient training diaries as well as physical therapist planning protocols. We will, in accordance with the current implementation literature, adopt measures to support the implementation process by; adapting the HiBalance program to the clinical context; engaging in shared-decision making with project clinician trainers; increasing trainer skills and by providing ongoing administrative and technical support [19].

#### Assessment of process outcomes

In order to fulfill the aims of the process evaluation which are to examine the quality and quantity of what was delivered it is necessary to assess outcomes such as the *fidelity* and *dose* of the program as it was delivered, as well as describe the *recruitment* process and *reach* of the intervention (Table 2) [21].

**Table 2:** Overview of the process evaluation involving process outcomes and barriers and facilitators, targeted groups and data collection methods

Process outcomes^a^	Targeted groups	Method of data collection
*Fidelity*	Patient training diaries/attendance Physical therapist trainers sessions plans	Assessment of group training protocols Focus group interviews
*Dose*	Physical therapist trainers (dose provided) Participants with PD (dose received)	Assessment of group training protocols Assessment of attendance at group training/home training protocols
*Recruitment*	Physical therapist trainers	Study logs and focus group interviews
*Reach*	Participants with PD	Study logs
Barriers and facilitators^b^	Targeted groups	Method of data collection
*Knowledge and beliefs* about the program *Relative advantage* of the program Need for *adaptability* of the program S*elf-efficacy* to train patients according to the programs core components Perceived *complexity*/difficulty of implementing the program in everyday practice	Physical therapist trainers	Focus group interviews
*Patients need and resources* *Knowledge and beliefs* about the balance program Perceived *complexity* of performing the training	Participants with PD	Structured questionnaire

^a^Guided UK Medical research council guidelines. ^b^Guided by constructs defined by Consolidated Framework for Implementation Research

**Fidelity** is defined as the degree to which the intervention is implemented as prescribed or intended by the program developers [34]. We aim to perform a fidelity assessment of the implementation process by monitoring the delivery of the core components of the HiBalance program, as it was designed. Adherence to these core components as well as the week-by-week structure of the sessions will be quantitatively assessed through collection and analysis of the planning materials used by trainers to plan the group training sessions (Additional file 1). Trainers at each clinic will be advised to dedicate approximately 20 min to planning training sessions, inspired by program materials, and to document training protocols following each session. Program features such as the warm-up phase of the training sessions, on the other hand, can be altered to achieve a better fit to specific clinics [19]. Fidelity will also be assessed qualitatively using open ended questions during focus-group interviews where trainers will be allowed describe this feature in their own words.

**Dose:** Both the dose of group training sessions delivered at the clinical sites, and the dose received by patients will be assessed. Dose received involves rate of participation in both group and home training sessions by participants. This data will be quantitatively assessed using attendance protocol as well as home training diaries.

**Recruitment:** the process of recruitment will be described quantitatively for the various clinics and trainers perceptions of recruitment will be qualitatively explored during the focus group interviews.

**Reach** refers to the extent to which the target audience comes into contact with the intervention [20]. We plan to describe to which extent those included in the study are representative of the target population of people with PD.

### Assessing barriers and facilitators to implementation (Aim III)

In addition to evaluating ‘how’ and ‘what’ was implemented by means of the process evaluation we will collect data concerning potential barriers and facilitators to the implementing the HiBalance program.

#### Research question relating to aim III

What are the potential barriers and facilitators to implementation of the HiBalance training program in outpatient clinical settings?

#### Determinant framework

The Consolidated Framework for Implementation Research (CFIR) will be used in the current study to guide the investigation of potential barriers and facilitators of the implementation process [35]. CFIR can be categorized as a determinant framework, in that it can be used to identify determinants (i.e. barriers and facilitators) at different levels (from the user to the program provider, to the organizational level) that can be thought to influence the implementation process [36]. CFIR provides a menu of constructs which are operationally defined and stem from various disciplines such as psychology and sociology [35]. We will choose constructs which we hypothesize to specifically impact the implementation of the HiBalance program from the following main domains defined by CFIR; Intervention characteristics; Inner setting; Outer setting and Characteristics of individuals. Table 2 provides an overview of the process outcomes as well as the subdomains of the CFIR constructs outlines above, which will guide data collection of the process evaluation and in the assessment of barriers and facilitators to implementation. However, we also acknowledge such frameworks do not conceptualize the role of unexpected events [37] and we aim therefore for flexibility in the data collection to allow for emergent factors concerning barriers and facilitators to be explored during the qualitative interview process [21].

#### Data collection

Data concerning aims II and III of the study will be collected using a mixed method approach. Focus group interviews will be performed with 3–6 physical therapists at the various clinical sites as a means to explore the process outcomes/barriers and facilitators outlined in Table 2. We will follow guidelines published by CFIR when developing the interview guide (http://cfirguide.org/tools.html). Additional file 2 demonstrates how interview questions are linked to CFIR constructs. Interviews will aim to gather detailed information regarding trainer experiences and perceptions throughout the evaluation period, in both the early and late stages of the process. Additionally, structured questionnaires will be used to capture aspects of patient experiences of the program upon completion of the 10-week training (Additional file 3).

#### Data analysis

Interviews transcripts will be systematically analyzed using thematic qualitative content analysis. This method is systematic, replicable and valid method in the analysis of text data [38]. During data analysis the research team will strive for an inductive approach to category development, by allowing categories to emerge from the data as opposed to driving category development by preconceived theory. The analysis will be performed systematically in steps [39], involved research group debriefing sessions which allow for researcher triangulation and ensure validity of the analysis process. Questionnaires and planning protocols will be analyzed primarily using descriptive statistics.

## Discussion

If evidence-based training programs are to benefit those with progressive neurological diseases, such as PD, the findings from RCT’s must be translated to and tested in everyday clinical settings. Additionally, there are a scarcity of studies which clearly report the underlying principles upon which such exercise programs are based [40]. The HiBalance program described in the current protocol has, on the other hand, been based on specific underlying theoretical principles and been shown effective in an RCT [17]. For these reasons this program is suitable for testing in an effectiveness study with the aim of further implementation on a wider scale. We find to date, no previously published trials which have tested the effectiveness of training interventions in PD while simultaneously evaluating the way in which the program was implemented.

By using a hybrid design we intend to fill gaps in the current knowledge concerning not only *what* to implement but also *how* to implement evidence-based balance training for mild to moderate PD in clinical practice. This study will therefore determine whether the adapted HiBalance program is effective at improving balance control, gait and physical activity level when provided in standard physiotherapy practice. Our results will also indicate whether implementation of the program is feasible on a wider scale in the Swedish context and what potential barriers and facilitators to this process may be. In this study protocol we use a theoretical approach to outline the core elements of the implementation process. We also hypothesize that results from our study will be highly relevant for those working within the field of neurological rehabilitation when considering ways in which to translate research findings into routine clinical care. Use of predefined constructs regarding the planned process evaluation also strengthens our design by enabling the easy comparison of our results with those of other studies concerning the implementation of healthcare interventions.

There will also be possible limitations to the proposed study. Firstly, this intervention does not consist of a fixed program of balance exercises, but relies on trainers’ adherence to the basic exercise principles in order to plan and adapt training sessions. While this feature allows exercises to be adapted and progressed at the individual level, it may be difficult to fully evaluate program fidelity. Extra efforts will therefore need to be made to evaluate the level of fidelity to the theoretical principles of the program throughout the implementation process. Secondly, randomization of participants into treatment and control groups was not considered feasible as the program is provided at the clinical sites as a part of standard physiotherapy treatment. Although consecutive inclusion of participants mirrors standard practice and therefore benefits external validity, a non-randomized design limits our ability to rule out the effects of selection bias between the control and intervention groups. Thirdly, in current physiotherapy treatment of people with PD in Sweden, there is no uniform standard care with which to compare the testing of this intervention. This may hamper the extent to which a standardized control group can be recruited.

This study represents a unique and structured approach to investigating the ways in which a rehabilitation intervention with proven efficacy responds to testing and implementation in real life clinical settings. Our study design should benefit people with progressive neurological conditions such as PD by providing valuable information which can speed up the process of translating research findings to routine health care. We hypothesize that study findings will produce highly applicable results not only for those in the field of PD, but to clinicians on a wider scale who are planning to translate research findings from training programs with proven effectiveness to the patients who need them.

### Trial status

At the time of manuscript submission the trial is in an ongoing phase of recruitment and data collection at the various clinical sites.

## Additional files

Additional file 1: Group training protocol. (DOCX 18 kb)
Additional file 2: Interview guide focus groups. (DOCX 48 kb)
Additional file 3: Patient questionnaire. (PDF 78 kb)
Additional file 4: Funding I and II (Swedish). (ZIP 1271 kb)
Additional file 5: Ethical approval (swedish). (PDF 81 kb)

## Abbreviations

CFIR: Consolidated framework for implementation research
PD: Parkinson’s disease
RCT: Randomized controlled trial
