# Supplementary material for: Evaluation and implementation of highly challenging balance training in clinical practice for people with Parkinson’s disease: protocol for the HiBalance effectiveness-implementation trial
Source: BMC Neurol. 2017 Feb 7;17:27. doi: 10.1186/s12883-017-0809-2 (PMC5297172; doi:10.1186/s12883-017-0809-2)
Supplement: Additional file 1: — Group training protocol. (DOCX 18 kb) [file 12883_2017_809_MOESM1_ESM.docx]

| **Group training report** | | | | | | **Date:______________**  **Name:___________________________**  **Clinic:___________________________** | | | | |
| --- | --- | --- | --- | --- | --- | --- | --- | --- | --- | --- |
| **Session plan: (session nr _/_)** | | | | | | | | | | |
| **Training performed** | |  | | | **Balance components** | | | | |  |
|  | **Description of exercise** | | **Standing** | **Walking** | **Stability**  **limits** | | **Antici-pation** | **Motor**  **agility** | **Sensory integration** | **Time period (min)** |
| **Exercise 1.** |  | | **□** | **□** | **□** | | **□** | **□** | **□** |  |
| **Exercise 2.** |  | | **□** | **□** | **□** | | **□** | **□** | **□** |  |
| **Exercise 3.** |  | | **□** | **□** | **□** | | **□** | **□** | **□** |  |
| **Exercise 4.** |  | | **□** | **□** | **□** | | **□** | **□** | **□** |  |
| **Exercise 5.** |  | | **□** | **□** | **□** | | **□** | **□** | **□** |  |

| Individual Assessment: | |  |  | | | |
| --- | --- | --- | --- | --- | --- | --- |
| : | : | : | **:** | : |  |  |
| To keep in mind for next training session: | | | | | | |
