# Supplementary material for: Evaluation and implementation of highly challenging balance training in clinical practice for people with Parkinson’s disease: protocol for the HiBalance effectiveness-implementation trial
Source: BMC Neurol. 2017 Feb 7;17:27. doi: 10.1186/s12883-017-0809-2 (PMC5297172; doi:10.1186/s12883-017-0809-2)
Supplement: Additional file 2: — Interview guide focus groups. (DOCX 48 kb) [file 12883_2017_809_MOESM2_ESM.docx]

**Objectives** of the interviews are to explore physiotherapist trainers’ perceptions and experiences of their involvement in HiBalance training – to explore facilitators and barriers to implementing the program in clinical practice as well as capture trainers eventual suggestions for adaptation to the program itself or the implementation process.

**Introduction**

Hello and thank you all for coming today. You are all here because you have expressed an interest in participating in this focus group interview. The purpose of this interview is for us to learn about your views on how the process of implementing the HiBalance program has gone up until now at your clinics. This information is crucial for us to be able to evaluate how we work, which aspects of the program/implementation are working well, and which areas need more attention or adaptation in order to ensure that we can successfully implement the program in the future.

Your participation is voluntary, so you may feel free to choose not to answer certain questions if you should choose. If everyone agrees to it, the interviews will be recorded so as to enable us to analyze them as text at a later stage. My role is to act mostly as moderator and there are no right or wrong answers to the questions I may pose. Feel free to be as critical as you choose. You may also chose to withdraw from participating at any stage.

I would also like to point out that no names will appear on the interview transcripts during our analysis, but what is said will be linked to a number in order to ensure confidentiality of what is said. Additionally, any personal references or names mentioned during the interviews will also be removed. Audio files will be saved on our servers and not be accessible to those working outside the project.

**Opening theme: Experiences of the HiBalance program**

- How would you **describe your experiences** so far of being involved in this research project involving the testing of the HiBalance program?
- What is it that motivates you to continue to train to HiBalance program/your involvement in the project?

**Theme I: Knowledge and beliefs concerning the program**

This theme will explore the CFIR construct: *Intervention characteristics,* of which we will explore the following subdomains; complexity; evidence strength and quality and relative advantage

- How complicated or difficult is it, according to your experience to plan and perform the training sessions?
- How do you experience the HiBalance program **when you compare** it to other methods of training balance in a group form with people with Parkinson’s disease (PD) or other neurological diagnosis?
- Do you think that the program, in its current form, will be an **effective method** of improving balance among the patients with PD in your group?
- What are your impressions concerning **the evidence** that is available regarding how effective or not, the HiBalance program is among the types of patient with Parkinson’s which you meet in your clinical practice?
- How, if at all, **does this knowledge affect your opinions** of the program and how it can be used?
- What kind of evidence do you think is necessary, concerning the effect of the program in clinical practice, **before it can be spread** and implemented on a wider scale in more clinics working with people with PD?

**Probes**

- What are the advantages and disadvantages of choosing this method?
- To what extent, if any, does the program depart from the workings of your regular clinical practice?
- Do you have any suggestions for ways which we could work to inform you better concerning the existing evidence of the program

**Theme II: Barriers to training according to the HiBalance method**

This theme will explore barriers experienced during the various stages of the program which can be related to the CFIR constructs: *Inner setting:* compatibility with clinical workflow and implementation climate- extent to which participation is encouraged/rewarded among decision makers within the clinical setting.

- Have you experienced any problems or **difficulties with planning and recruiting for the program** prior to the training period which you have just completed?
- Have you experienced any problems or difficult situations during the training sessions which have meant that you have **had to depart from your training plan?**
- Have you experienced any other problems which have arisen as a result of **being involved in a research project** which have caused difficulties for you in your everyday clinical work?

**Probes**

- Can you give any examples of these kind of difficult situations?
- What do you think these difficulties were caused by?
- Could they have been foreseen, or prevented in any way?
- Is there anything that you would have needed in connection with the planning, training or assessment of the intervention, that you didn’t receive?

**Theme III: Adaptability of the program**

This theme will explore the CFIR construct: Intervention characteristics: adaptability

- When you look back on how you have worked, **have you made any changes** to the program, from lessons that you have learned from your involvement?
- What kind of changes or **refinements,** if any, do you think **you will need to make** to the program so it will work effectively at your clinic?
- Do you think you will be able to make these changes? And why or why not?
- Are there components of the program or process of implementing it that should **not be changed**, in your opinion? Which components are these?

**Theme IV: Facilitatory factors/what has worked well**

- If you look back on the group training sessions that you have held, which aspects of the program have felt **positive for patient** and/or for yourselves?
- Can you give examples other factors/ situations which have led to training sessions which have felt successful?
- What aspects of the training do you **value the most**? Are there parts of the program that you find especially motivating, where you feel- this is good for my patients?

**Theme V: Access to information, knowledge and support.**

This theme will explore the CFIR construct: *Inner setting*: Access to knowledge and information

- What are your opinions on the **training that you received** regarding HiBalance?
- What was the **quality of the materials** or information regarding the HiBalance program that you received?
- Is there any kind of material/ resources that you would have needed but **didn’t receive** which would have eased your participation?
- What kind of **support is necessary to receive** as a trainer of this program, in order to aid participation in the project?

**Probes**

- How were written resources? Were they timely/ relevant/ sufficient?
- How was the personal contact with the research group?
- Is there some kind of support that you need more of? If so, in which form would you like to receive it?

**Theme VII: Patients needs**

This theme will explore the CFIR construct: *Outer setting*: Patients needs and resources

- How well do you think that the program meets the **needs** of the patients?
- What kind of **barriers** do you think that patients will experience when participating in the program?
- Which **type of patient** with PD do you think is most suitable to gaining from this program, and what type of patient do you think is unsuitable for this particular approach?

**ii) Trainer experiences, knowledge and needs**

This theme will explore the subdomains of self-efficacy and reflecting and evaluating from different CFIR constructs *Characteristics of individuals* and *Process*, respectively.

- How **confident are you** that you can train the program as it is described?
- What gives you that level of confidence (or lack of confidence)?
- What kind of **feedback reports**, if any, about the implementation or the program itself would you like to receive from the research group?

**Probes**

- Has your confidence changed during the training period?
- How many training periods do you think that you need to participate in in order to feel confident in your delivery of the program?
- In what form would you like to receive feedback about details of the project, and at what time intervals?

**Rounding off**

Do you feel as if you have gotten the chance to voice your opinions concerning your practical experiences and thoughts on the HiBalance program?

Is there anything which I haven’t asked about which you would like to add before we finish our discussion?
