# Supplementary material for: Evaluation and implementation of highly challenging balance training in clinical practice for people with Parkinson’s disease: protocol for the HiBalance effectiveness-implementation trial
Source: BMC Neurol. 2017 Feb 7;17:27. doi: 10.1186/s12883-017-0809-2 (PMC5297172; doi:10.1186/s12883-017-0809-2)
Supplement: Additional file 3: — Patient questionnaire. (PDF 78 kb) [file 12883_2017_809_MOESM3_ESM.pdf]

1. [Last name], [First name]

1. How has the training program affected your balance?

- ☐ My balance is a lot better
- ☐ My balance is somewhat better
- ☐ My balance is unchanged
- ☐ My balance has worsened

2. To what extent do you think the level of difficulty of the exercises increased successively during the training period?

- ☐ To a very small extent
- ☐ To a small extent
- ☐ Partly
- ☐ To a large extent
- ☐ To a very large extent

3. Mark the circle which most represents your opinion of how the following exercises challenged your balance during group training.

	To a very small degree	To a small degree	Partly	To a large degree	To a very large degree	Don't know
Stretching/rotation exercises i standing	☐	☐	☐	☐	☐	☐
Throwing/kicking a ball in standing	☐	☐	☐	☐	☐	☐
Standing exercises on soft/unstable surfaces	☐	☐	☐	☐	☐	☐
Walking exercises on soft/unstable surfaces	☐	☐	☐	☐	☐	☐
Walking exercises involving carrying a ball, tray or other item	☐	☐	☐	☐	☐	☐
Walking over or around obstacles	☐	☐	☐	☐	☐	☐
Walking exercises involving simultaneous counting or word tasks	☐	☐	☐	☐	☐	☐

Your own example of an exercise which you thought was especially chalenging:

4. Have you experienced increased fatique which you associated with participation i the program?

☐ Not at all

☐ Rarely

☐ Sometimes

☐ Fairly often

☐ Very often

5. Have you since participating in the program experienced increased pain which you attribute to the training, which has lasted more than 48 hours?

☐ Yes ☐ No

If yes, describe when, where and how often you experienced this pain

6. Have you, since participation in the program, reduced other everyday activities which you would have liked to have undertaken due to tiredness or pain?

☐ Yes

☐ No

If yes, describe which activites and how often:

7. Since participating in the program, have you commenced or resumed any activity which you have previously considered difficult to do?

☐ Yes

☐ No

If yes, describe which activity:

8. Was there anything which you thought was missing from the group-based balance training program?

☐ Yes

☐ No

If yes, describe what you thought was missing:

---

9. Have you completed the home exercise program during the entire 10-week period?

☐ Yes

☐ No

If yes, describe how many times a week you have performed the home exercise program

---

10. The following statement concern the home exercise program, mark the circle which most represents your experiences

	Strongly disagree	Disagree	Agree	Strongly agree	Don't know
The exercises were in line with my capacity	☐	☐	☐	☐	☐
I felt that the home exercises became gradually easier to perform	☐	☐	☐	☐	☐
The home exercise program felt meningful to carry out	☐	☐	☐	☐	☐
I am motivated to continue carrying out the home exercises on my own when the training period is finished	☐	☐	☐	☐	☐

11. Do you think that your balance was challenged during the group training sessions?

- [ ] To a very small extent
- [ ] To a small extent
- [ ] Partly
- [ ] To a large extent
- [ ] To a very large extent

12. Was there a particular exercise in the home exercise program which was especially difficult to do?

- [ ] Yes
- [ ] No

If yes, describe which exercise:

13. Did you slip, trip or loose you balance so that you fell during the home exercise program?

- [ ] Yes
- [ ] No

If yes, describe how many times this happened and if you injured yourself in any way:

14. Was there anything you thought was missing from the home exercise program?

- [ ] Yes
- [ ] No

if yes, describe what you think was missing
