# Supplementary material for: Evaluation and implementation of highly challenging balance training in clinical practice for people with Parkinson’s disease: protocol for the HiBalance effectiveness-implementation trial
Source: BMC Neurol. 2017 Feb 7;17:27. doi: 10.1186/s12883-017-0809-2 (PMC5297172; doi:10.1186/s12883-017-0809-2)
Supplement: Additional file 4: — Funding I and II (Swedish). (ZIP 1271 kb) [file 12883_2017_809_MOESM4_ESM.zip › Additional file 5i) Funding I_Swedish (original)R1.pdf]

VÅRDALSTIFTELSEN

Franzén
Vårdal.

Stockholm 14-11-12 Dnr-2014-0079

**Kontrakt för projekt inom Vårdalstiftelsen**

Detta kontrakt är upprättat mellan anslagsmottagaren Erika Franzén och Vårdalstiftelsen.
Kontraktet är upprättat i tre exemplar, ett till vardera parten samt ett till anslagsförvaltaren.
Kontraktet avser anslag till projektet ***"Improving balance and physical activity in elderly with Parkinson's disease –efficacy and implementation of the HiBalance program"***- och anger villkor för medelstilldelningen.

Medlen är avsedda för att genomföra projektet i enlighet med
– Allmänna villkor (bifogas detta kontrakt)
– Inlämnad ansökan.

För ändamålet har Vårdalstiftelsens styrelse beviljat medel om totalt *3 000 000* kronor.
Medlen inkluderar förvaltningsavgifter, moms och ev. andra påslag med maximalt 20 %
och fördelas enligt följande:

Anslagsperiod: 1 januari 2015- 31 december 2015: 1 000 000 kronor
Anslagsperiod: 1 januari 2016- 31 december 2016: 1 000 000 kronor
Anslagsperiod: 1 januari 2017- 31 december 2017: 1 000 000 kronor

Medel beviljas under förutsättning att projektet inte erhåller full finansiering från annat håll och får endast användas för kostnader i enlighet med ansökan.

Projektet skall ha startat senast tre månader från första utbetalningen.
Om så inte skett har Vårdalstiftelsens styrelse rätt att dra tillbaka anslaget med omedelbar verkan.

Medlen betalas ut vid två tillfällen enligt följande:
December 2014 utbetalas 1/3 av totalt beviljat belopp. ✓
Mars 2015 utbetalas 2/3 av totalt beviljat belopp. ✓

Medlen får disponeras högst 6 månader efter anslagsperiodens slut. tom 2018-06-30.

Ytterligare förlängning av dispositionstiden kan eventuellt medges efter skriftlig begäran.

Vetenskaplig och ekonomisk slutrapportering skall ske enligt bestämmelser i de allmänna villkoren. senast 2018-12-31.

Eventuellt återstående medel skall efter dispositionstidens slut återföras till Vårdalstiftelsens plusgirokonto 72 28 63–8.

1 milj Bokf 8/1-2015, Period 2014.12.

För Vårdalstiftelsen

.............................................................................

Thorbjörn Larsson
VD Vårdalstiftelsen

2 milj Bokf 16/3-2015.

Dnr 4-3603/2014

Proj H1454 73 133

**Ifylls av anslagsmottagaren**

Jag förklarar mig härmed beredd att ta emot anslaget och förbinder mig att följa med anslaget förbundna villkor som anges i kontraktet samt i allmänna villkor enligt bilaga ~~A till detta~~ kontrakt.

2014-11-23 ........................................ Datum

........................................ Underskrift

**Ifylls av anslagsförvaltaren (rektor/ekonomiskt ansvarig)**

BG 5310-6407
........................................
Plusgiro kontonr/ Bankkontonummer

........................................
Internkontonr

KAROLINSKA INSTITUTET
........................................
Universitet/Högskola

Vi förbinder oss att förvalta anslaget i enlighet med kontraktets bestämmelser

20141125
........................................
Datum

........................................
Underskrift

KI
........................................
Universitet/Högskola

........................................
Namnförtydligande

KAROLINSKA INSTITUTET ANNO 1810
Maria Eriksdotter
Prefekt
Institutionen för Neurob[illegible]
vårdvetenskap och sam[illegible]

08-524 86479
........................................
Kontakt, telefon alt. e-mail.
