# Supplementary material for: Evaluation and implementation of highly challenging balance training in clinical practice for people with Parkinson’s disease: protocol for the HiBalance effectiveness-implementation trial
Source: BMC Neurol. 2017 Feb 7;17:27. doi: 10.1186/s12883-017-0809-2 (PMC5297172; doi:10.1186/s12883-017-0809-2)
Supplement: Additional file 4: — Funding I and II (Swedish). (ZIP 1271 kb) [file 12883_2017_809_MOESM4_ESM.zip › Additional file 5ii) Funding II_Swedish (original)R1.pdf]

FORTE:

Forskningsrådet för
hälsa, arbetsliv och välfärd

4-3004/2014

2014-11-24

Erika Franzén
Karolinska Institutet
Inst för neurobiologi, vårdvetenskap och
samhälle (NVS), Fysioterapi
Fack 23100, Alfred Nobels Allé 23
141 83 HUDDINGE

BESLUT

2014-4764 **Åldrande: Improving balance and physical activity in elderly with Parkinson's disease – long-term efficacy and effectiveness in a clinical practise of the HiBalance program**

Projektledare: Erika Franzén, Karolinska Institutet, Inst för neurobiologi, vårdvetenskap och samhälle (NVS), Fysioterapi
Projektets hemvist: Karolinska Institutet, Inst för neurobiologi, vårdvetenskap och samhälle (NVS), Fysioterapi
Medelsförvaltare: Karolinska Institutets ekonomienhet, bg 5321-9416

Forskningsrådet för hälsa, arbetsliv och välfärd, Forte, har den 17 november 2014 beslutat att för ovanstående projekt bevilja 3 230 000 kronor med följande fördelning:

2014	600 000
2015	1 400 000
2016	1 230 000

Det beviljade beloppet inkluderar indirekta kostnader och ett Open Access publiceringsbidrag på 30 000 kronor (år 2016). Användningen av publiceringsbidraget kommer att följas upp av Forte och bör därför redovisas separat. För mer information om Fortes Open Access policy hänvisas till http://www.forte.se/sv/Soka-bidrag/Open-access/.
Det beviljade beloppet kommer att betalas ut till medelsförvaltaren enligt bifogad utbetalningsplan. För att medlen ska betalas ut måste ett undertecknat godkännande av villkoren ha kommit in till Forte. För medlen gäller bifogade generella villkor, fastställda 2013. Därtill gäller följande särskilda villkor:

- Godkännandet ska vara oss tillhanda senast **2014-12-09**
- Populärsammanfattning på svenska och engelska ska lämnas senast 2014-12-09 i eKlara
- Lägesrapport ska lämnas senast 2016-02-28
- Dispositionstid t o m 2018-11-30
- Slutredogörelse med ekonomisk redovisning ska lämnas senast 2019-02-28
- Godkännande av regional etikprövningsnämnd skall insändas till Forte.

Separata anvisningar för återrapportering i det nya Prisma-systemet skickas ut vid senare tillfälle.

Forskningsrådet för hälsa, arbetsliv och välfärd

Peter Allebeck
Huvudsekreterare

Therese Woodhill
Forskningssekreterare

Swedish Research Council for Health, Working Life and Welfare

Address Box 894, SE-101 37 Stockholm
Phone +46 8 775 40 70 **Fax** +46 8 775 40 75
Org.nr 202100-5240 **E-mail** forte@forte.se **www.forte.se**

H1454 73 123

GODKÄNNANDE

2014-4764
Therese Woodhill

**2014-4764**	**Åldrande: Improving balance and physical activity in elderly with Parkinson's disease – long-term efficacy and effectiveness in a clinical practise of the HiBalance program**
Projektledare:	Erika Franzén, Karolinska Institutet, Inst för neurobiologi, vårdvetenskap och samhälle (NVS), Fysioterapi
Projektets hemvist:	Karolinska Institutet, Inst för neurobiologi, vårdvetenskap och samhälle (NVS), Fysioterapi
Medelsförvaltare:	Karolinska Institutets ekonomienhet, bg 5321-9416

De i Fortes beslut av den 17 november 2014 angivna villkoren för bidraget godkännes:

....
Erika Franzén
Projektledare

....
Underskrift av behörig företrädare för
Karolinska Institutets ekonomienhet

[illegible] Neurobiologi, [illegible] samhälle.
Namnförtydligande

Maria Eriksdotter
Head of Department
Department of Neurobiology,
Care Sciences and Society

Ett undertecknat godkännande insändes till Forte, Box 894, 101 37 Stockholm, så att det är oss tillhanda senast 2014-12-09.

Forte **UTBETALNINGSPLAN**

Diarienr	**2014-4764**
Titel	Åldrande: Improving balance and physical activity in elderly with Parkinson's disease – long-term efficacy and effectiveness in a clinical practise of the HiBalance program
Projektledare	Erika Franzén<br>Inst för neurobiologi, vårdvetenskap och samhälle (NVS), Fysioterapi<br>Karolinska Institutet
Projekttid	2014-12-01 -- 2017-11-30
Medelsförvaltare:	Karolinska Institutets ekonomienhet

Beviljat		
	2014	600 000
	2015	1 400 000
	2016	1 230 000
	2017	0

**Status**	**Datum**	**Belopp**	**Utbetalt**	**Utbet.datum**	**Ver.nr.**
OK	2014-12-25	600 000			
OK	2015-01-25	116 666			
OK	2015-02-25	116 666			
OK	2015-03-25	116 666			
OK	2015-04-25	116 666			
OK	2015-05-25	116 666			
OK	2015-06-25	116 666			
OK	2015-07-25	116 666			
OK	2015-08-25	116 666			
OK	2015-09-25	116 666			
OK	2015-10-25	116 666			
OK	2015-11-25	116 666			
OK	2015-12-25	116 674			
OK	2016-01-25	102 500			
OK	2016-02-25	102 500			
OK	2016-03-25	102 500			
OK	2016-04-25	102 500			
OK	2016-05-25	102 500			
OK	2016-06-25	102 500			
OK	2016-07-25	102 500			
OK	2016-08-25	102 500			
OK	2016-09-25	102 500			
OK	2016-10-25	102 500			
OK	2016-11-25	102 500			
OK	2016-12-25	102 500			
	**Beviljat**	**Summa i plan**	**Summa utbetalt**	**Återstår av betalplan**	
	3 230 000	3 230 000	0	3 230 000	

B,G,UUTB Under utbetalning UTB Utbetald OK Att betalas ut INB Inbeta

Forskningsrådet för hälsa, arbetsliv och välfärd STY 2014-11-17 Beslutsunderlag 2014-4764

Handläggare: Therese Woodhill
Bedömningsgrupp: Åldrande 2014

Erika Franzén, Karolinska Institutet, Inst för neurobiologi, vårdvetenskap och samhälle (NVS), Fysioterapi
Åldrande: Improving balance and physical activity in elderly with Parkinson's disease – long-term efficacy and effectiveness in a clinical practise of the HiBalance program

År	Sökt	Förslag:	Bevilja
2014	0		600 000
2015	647 348		1 400 000
2016	1 653 135		1 230 000
2017	1 201 050		0
Summa	3 501 533		3 230 000

Villkor: Godkännande av regional etikprövningsnämnd skall insändas till Forte.

Bedömning
Application reference number: 2014-4764
Project leader: Erika Franzén

Fear of falls in old age can lead to a decrease in mobility and physical activity and an increase in sedentary behavior. This decrease in physical activity leads to an increase in risk for falls (and, hence, fear of falling). Parkinson's disease (PD) affects many physiological systems essential for balance control. As a consequence, older people with Parkinson's disease have an increased risk for fear of falls and physical inactivity. In contrast to medication (which has little effects), trainings and exercise might be a more promising pathway.

This project is an extension of a preceding project which tested the short term effects of a new balance training in patients with Parkinson Disease (PD). The proposed project consists of two steps (which are called step 2 and step 3, these are the steps after the first step of testing short effects of the training). In step 2, the long-term effects of the balance program will be tested (n=50 for experimental and control group). In project phase "step 2", there is a standard experimental-control design with measurements before and after the treatment as well as after 6 and 12 months. In step 3, the effectiveness of the training in six different clinical settings will be tested. In project phase "step 3", the effectiveness of the training under clinical conditions will be tested in six settings with 50 individuals in two groups (experimental, control).

This proposal fits well into the topic "research on physical and mental disabilities and the combinations of preventative and rehabilitative measures that can help to promote the conditions needed for individuals to participate in active employment and lead active day-to-day lives" of the call "Ageing and health".

This is an highly relevant, carefully designed study on the long-term effects of a balance training which is theoretically well founded. The project's implications concern the improvement of the treatment for patients with Parkinson's Disease. In addition, the clinical trial attempts the implementation of the balance training in clinical settings.

Recommendation: The panel recommends funding.

Forskningsrådet för hälsa, arbetsliv och välfärd

Generella villkor för bidrag till forskning från Forskningsrådet för hälsa, arbetsliv och välfärd, Forte /i det följande Rådet/

Fastställda för Fortes del den 3 oktober 2013. Villkoren gäller för bidragsbeslut med utbetalning tidigast från och med den 1 december 2013. Villkoren gäller i den utsträckning annat inte anges i bidragsbeslutet.

Definitioner

Medelsförvaltare avser den juridiska person som förvaltar bidraget till forskning som Forte betalar ut. Forte godkänner nya medelsförvaltare efter prövning.

Projektledare avser den person som är ansvarig för ansökan (sökande) till Forte och som ansvarar för att planera och genomföra projektet.

Sökande avser person eller organisation som är ansvarig för ansökan till Forte. När en organisation är sökande till Forte har organisationen samma ansvar som en projektledare.

Projekt avser de verksamheter som stöds av forskningsbidraget.

Allmänt

Fortes beslut om bidrag blir giltigt när både medelsförvaltare och projektledare har bekräftat sina åtaganden enligt nedan.

Åtaganden

Fortes åtaganden

Rådet förbinder sig att betala ut beslutat bidrag till den medelsförvaltare och enligt den utbetalningsplan som anges i beslutet. Utbetalningen sker normalt månadsvis, med en tolftedel av det för resp. år angivna beloppet, om inte annat anges i beslutet. Utbetalning sker efter att beslutet blivit giltigt. De i beslutet angivna beloppen är definitiva. Kompensation för ökade lönekostnader eller andra kostnadsökningar ges inte.

Bidrag till forskningsprojekt vid universitet och högskolor ska omfatta medel för direkta och indirekta kostnader i samma proportioner som beräknats för projektet i sin helhet.

Beslut om bidrag gäller under förutsättning att Forte anvisas de medel som föreslås av regeringen.

Medelsförvaltarens åtaganden

Medelsförvaltaren åtar sig

- att i egenskap av arbetsgivare för projektledaren se till att denne kan disponera egen och andra anställdas arbetstid i den utsträckning som krävs för att projektet ska kunna genomföras i huvudsak överensstämmande med den projektplan som ingivits till rådet, liksom för att resultaten av projektet ska kunna publiceras,
- att svara för att projektledaren och annan av projektet berörd personal får tillgång till lokaler, utrustning och andra resurser som krävs för projektets genomförande,
- att tillse att den forskning som bedrivs inom projektet uppfyller de villkor och förutsättningar som anges i svensk lagstiftning,
- att tillse att den forskning som bedrivs inom projektet inte har kommersiella bindningar som påverkar dess objektivitet, oberoende eller öppenhet,
- att årligen till Forte rapportera uttag av direkta och indirekta projektrelaterade kostnader i enlighet med Fortes anvisningar,
- att förvalta bidraget enligt i beslutet angivna villkor, samt svara för administration av verksamheten.

Medelsförvaltaren kan efter samråd med projektledaren besluta att starten av projektet ska senareläggas eller att ett uppehåll i projektet ska göras, så länge den i beslutet angivna dispositionstiden inte överskrids. Detta kan bli nödvändigt till exempel för att projektledaren måste tas i anspråk för annan tjänstgöring eller

om annan nyckelpersonal inte kan disponeras för projektet och inte omedelbart kan ersättas.

Projektledarens åtaganden

Projektledaren (sökande) har det vetenskapliga ansvaret för projektet så som detta beskrivits i bidragsansökan till rådet med avseende på objekt och metod. I detta ingår att planera och genomföra projektet i huvudsaklig överensstämmelse med den till rådet ingivna planen och de villkor som anges i beslutet, dock med de justeringar som kan krävas om rådets bidrag understiger sökt belopp.

Projektledaren ansvarar för att erforderliga tillstånd och godkännanden finns. Då projektet innefattar forskning där människor eller försöksdjur medverkar ska projektledaren se till att det finns ett godkännande av regional etikprövningsnämnd och djurförsöksetisk nämnd i den utsträckning som följer av lag och av regeringen utfärdade föreskrifter. Därutöver ska projektledaren se till att etiska principer som följer av föreskrifter som rådet utfärdat efterlevs.

I det vetenskapliga ansvaret ingår också att se till att resultaten av forskningsarbetet publiceras i vetenskaplig tidskrift med internationell räckvidd eller görs tillgängliga på annat motsvarande sätt. Projektledaren är skyldig att se till att han/hon inte träffar avtal med kommersiell eller annan intressent som hindrar honom/henne att publicera resultatet av den forskning som bedrivs med stöd av bidraget från rådet, eller som fördröjer publiceringen i mer än två månader. Fördröjningen får dock uppgå till högst fyra månader om syftet är att möjliggöra en ansökan om patent, grundat helt eller delvis på ovan avsedda forskningsresultat.

Projektledaren ska garantera att forskningsresultaten finns tillgängliga för alla (Open Access) inom sex månader efter publicering. I de fall publiceringen sker som parallellpublicering i öppna institutionella arkiv, ska deposition ske vid publiceringstillfället och göras öppet tillgängligt inom sex månader. Open Accessreglerna gäller tills vidare vetenskapligt bedömda texter i tidskrifter och konferensrapporter.

Projektledaren ska också, i förekommande fall i anslutning till vad som redovisats i ansökan, se till att resultat av mer allmänt intresse sprids också till mottagare utanför vetenskapssamhället. Projektledaren ska vidare lämna tillgång till det fullständiga materialet för eventuell vetenskaplig granskning, om rådet så begär.

Vid spridning av resultat ska projektledaren ange att forskningen bedrivits med stöd av Forte. Vid publicering av vetenskapliga originalartiklar och avhandlingar ska diarienumret för forskningsbidraget anges under rubriken "acknowledgements".

Bidragsperiod

I beslutet anges för vilken tid bidrag ges (bidragsperioden), liksom hur länge bidraget får disponeras. Rådets, medelsförvaltarens och projektledarens åtaganden gäller från och med den dag samtliga nämnda godkänt villkoren. Åtagandena gäller till och med den dag då slutlig redovisning godkänts av rådet och eventuella outnyttjade medel återbetalats.

Dispositionsrätt

Av beslutet framgår under vilken period bidraget utbetalas. Om inte annat anges i beslutet får bidraget disponeras under nämnda period och därtill i ytterligare ett år, räknat från bidragsperiodens sista dag (dispositionstiden). Om särskilda skäl föreligger, kan förlängning av dispositionstiden beviljas. Begäran om detta ska lämnas in gemensamt av medelsförvaltaren och projektledaren i god tid före dispositionstidens utgång. Begäran ska åtföljas av uppgift om ännu inte utnyttjade medel, samt en översiktlig plan för slutförande av projektet.

Bidraget ska disponeras i huvudsaklig överensstämmelse med till rådet ingiven projektplan, dock med de justeringar som kan krävas om rådets bidrag understiger sökt belopp. Vidare ska bidraget disponeras enligt villkoren i beslutet. För mer betydelsefulla ändringar som gäller bidragets disposition eller beslutsvillkoren krävs en skriftlig framställning till rådet från medelsförvaltaren och projektledaren. Forte kan bevilja ändringen efter prövning.

Bidraget får inte användas till stipendier som är avsedda att vara ett alternativ till lön eller annan studiefinansiering för doktorander.

Arbetsgivarförhållanden och anställningsvillkor

Projektledaren ska vara anställd av medelsförvaltaren, om inte rådet och medelsförvaltaren kommer överens om annat. Om projektledaren har en annan arbetsgivare ska denne godkänna hans/hennes åtaganden.

Utrustning

Medelsförvaltaren har äganderätten till utrustning och andra inventarier som anskaffas till ett projekt med stöd av bidrag från rådet. Utrustningen ska dock disponeras för projektet så länge projektet pågår.

Skyldighet att informera om andra bidrag

Sökanden ska anmäla eventuella ansökningar till andra finansiärer avseende medel för samma eller liknande ändamål. Detsamma gäller för redan erhållna bidrag från andra finansiärer. Om bidrag från annan finansiär erbjuds eller erhålls för samma eller liknande ändamål under forskningsprojektets gång, ska projektledaren snarast anmäla detta till rådet. Det ska i redovisningen till rådet särskilt framgå i vilken utsträckning denna andra finansiär kan påverka genomförande, analys, tolkning och redovisning av resultaten. Dessutom ska det framgå vem som kommer att förfoga över resultaten. Rådet förbehåller sig rätten att ompröva bidragsbeslut baserat på information om andra bidrag.

Medelsförvaltaren får i samråd med projektledaren besluta att del av erhållet bidrag från rådet används som finansiering av medverkan (så kallad motfinansiering) i av EU stött projekt inom samma område eller med samma inriktning som det projekt som bidraget från rådet avser.

Rapportering

Medelsförvaltaren ansvarar för att tillhandahålla de uppgifter Forte efterfrågar i samband med uppföljning och utvärdering av forskningen, såväl under pågående som efter avslutad bidragsperiod. Redogörelser och rapporter avseende projektet ska lämnas i den ordning som anges i bidragsbeslutet eller när rådet särskilt begär det. En av rådet utsedd revisor eller annan granskare har rätt att granska den bokföring som hör till beslutet. Denne har härvidlag rätt till fullständig insyn i projektet, bland annat rätt att få kopior av samtliga verifikationer avseende utgifter och inkomster som hänför sig till projektet.

Ekonomisk slutredovisning

Medelsförvaltaren ska lämna ekonomisk redovisning enligt Fortes anvisningar. Slutredovisning ska lämnas senast tre månader efter avstämningsdatum, vilket normalt är detsamma som sista dag för dispositionstiden. Forte kan komma att avföra ansökan om nytt bidrag om medelsförvaltaren inte lämnat slutredogörelser för tidigare beviljade bidrag inom utsatt tid. Rådets ovan beskrivna rätt till revision av redovisningen gäller även den ekonomiska slutredovisningen.

Rådet kan besluta att den ekonomiska redovisningen ska lämnas vid annan tidpunkt. Sådant beslut ska ange avstämningsdag och delges medelsförvaltaren minst tre månader före avstämningsdagen.

Om ett projekt avbryts i förtid gäller särskilda bestämmelser som anges nedan.

Återbetalning av överskott

Outnyttjade bidrag som redovisas i inlämnad ekonomisk slutredovisning ska återbetalas till Forte av medelsförvaltaren inom 30 dagar från inlämningsdatum. Om överskottet understiger ett halvt prisbasbelopp får det emellertid behållas och användas för forskningsändamål liknande det som bidraget avsåg. Är överskottet större ska det i sin helhet återbetalas till Forte.

Förtida avslutande av projekt

Svårigheter att genomföra projektet - allmänt
Om det projekt som rådet beviljat bidrag till inte kan genomföras enligt plan och avvikelsen inte är obetydlig ska medelsförvaltaren omgå-

ende underrätta rådet om detta. Detsamma gäller om utrustning, för vars inköp rådet beviljat medel, inte kan anskaffas. Härvidlag ska också redovisas hur det projekt påverkas för vilket utrustningen avsetts. Rådet och medelsförvaltaren förutsätts efter samråd med projektledaren komma överens om hur den uppkomna situationen ska hanteras.

Svårigheter att genomföra projektet på grund av förändrade förhållanden för projektledaren
Om projektledaren på grund av dödsfall, långvarig sjukdom, övergång till annan arbetsgivare eller av annat skäl inte längre kan genomföra projektet ska medelsförvaltaren omedelbart anmäla detta till rådet. Beslut om projektet ska erhålla fortsatt finansiering eller avslutas fattas av Forte efter samråd med medelsförvaltaren, och där så är möjligt med projektledaren. Om projektet avslutas, ska medelsförvaltaren återbetala ännu inte disponerade medel som är avsedda för projektet.

Om skälet till förändringen är att projektledaren övergår till anställning vid annan medelsförvaltare, kan rådet besluta att projektet flyttas till denna andra medelsförvaltare och att det drivs vidare med samma projektledare. Innan sådant beslut fattas ska samråd ske med berörda medelsförvaltare om villkor för flyttning.

Kostnader för förtida avveckling av ett projekt regleras enligt nedan.

Brott mot gjorda åtaganden
Om rådet eller medelsförvaltaren eller projektledaren i väsentligt avseende bryter mot beslutsvillkoren och inte inom en månad (30 dagar) efter skriftlig anmaning upphör härmed, kan rådet eller medelsförvaltaren avsluta sina åtaganden med omedelbar verkan.

Följande omständigheter i anslutning till forskning utgör grund för beslut av rådet att avbryta utbetalning av ett bidrag:

- oriktiga uppgifter i ansökan, eller utelämnande av uppgifter som krävs,
- underlåtenhet att under projektets gång rapportera sådana nya omständigheter som enligt beslutsvillkoren ska rapporteras,
- ekonomiska oegentligheter,
- oetiskt handlande,
- kommersiella bindningar som påverkar projektets objektivitet, oberoende eller öppenhet,
- projektledaren har genom annat handlande visat sig klart olämplig att uppbära bidrag från rådet,
- underlåtenhet att inlämna återrapportering enligt Fortes anvisningar.

Utrustning
Vid förtida avveckling av ett projekt kan rådet efter samråd med berörda medelsförvaltare besluta att utrustning som anskaffats med stöd av rådets bidrag ska överlämnas till rådet eller till annan medelsförvaltare.

Ekonomisk slutredovisning vid förtida avslutande av projekt
Om rådet beslutar att avbryta utbetalning av ett bidrag eller om ett projekt avbryts i förtid av andra skäl ska ekonomisk slutredovisning av projektet ske senast tre månader efter den dag parterna kommit överens om att projektet ska upphöra eller då endera av parterna ensidigt fattat sådant beslut. Rådet ska ställa krav på återbetalning av erhållet och utbetalat bidrag till medelsförvaltaren inom tre månader (90 dagar) efter att rådet fått den ekonomiska slutredovisningen.

Kostnader för förtida avveckling av projekt
Om rådet beslutar att avbryta utbetalning av ett bidrag och detta inte beror på brott mot villkoren från medelsförvaltarens sida förutsätts medelsförvaltaren och rådet komma överens om hur skäliga avvecklingskostnader ska finansieras.
