# Supplementary material for: Evaluation and implementation of highly challenging balance training in clinical practice for people with Parkinson’s disease: protocol for the HiBalance effectiveness-implementation trial
Source: BMC Neurol. 2017 Feb 7;17:27. doi: 10.1186/s12883-017-0809-2 (PMC5297172; doi:10.1186/s12883-017-0809-2)
Supplement: Additional file 5: — Ethical approval (swedish). (PDF 81 kb) [file 12883_2017_809_MOESM5_ESM.pdf]

Regionala etikprövningsnämnden i Stockholm
Protokoll 2016/2:2

Utdrag ur protokoll från sammanträde den 2 mars 2016 i avdelningen 2.

Diarienummer:
2016/201-31/2
Föredragande:
Yvonne Forsell

Sökande: Karolinska Institutet
Behörig företrädare: Maria Eriksdotter
Projekt: Implementering av HiBalance träning för personer med Parkinsons sjukdom
Forskare som genomför projektet: Erika Franzén

BESLUT

Nämnden godkänner forskningen med följande villkor.

1. I forskningspersonsinformationen ska individen tillfrågas om att delta i stället för att hälsas välkommen.
2. Kontaktperson för PUL saknas i forskningspersonsinformationen.

Hur man överklagar, se särskild information.

Beslut expedierat till behörig företrädare och ansvarig forskare.

Ordförande
Christian Groth
Ledamöter med vetenskaplig kompetens
Pär Sparén vetenskaplig sekreterare *(medicinsk epidemiologi)deltog inte i 2016/191 och 2016/192 pga. jäv*
Aniko Bartfai (*klinisk neuropsykologi*)
Mats Blennow *(pediatrik)deltog inte i 2016/202 pga. jäv*
Kristian Borg *(neurologi)skriftliga föredragningar*
Mats Eriksson *(endokrinologi) deltog inte i 2015;2294,2325,1954,2016;175;181*
Gunilla Sandborgh *(odontologi)*
Yvonne Forsell (*psykiatri, geriatrik*) *deltog inte i 2016/175 pga. jäv*
Greger Lindberg (*gastroenteriologi*)
Thomas Sejersen *(neuropediatrik) deltog inte i 2016/242, 250, 264*

Ledamöter som företräder allmänna intressen
Jörgen Bengtsson
Kemo Ceesay
Gunilla Johansson
Ewa Schenström
Ingmar Wallén

Administrativ sekreterare
Jenny Karte

§ **1** Ordföranden förklarar sammanträdet öppnat.

§ **2** Greger Lindberg förordnas som vetenskaplig sekreterare i ärende 2016/191 och 2016/192 då Pär Sparén är jävig.

§ **3** Ansökningar om etisk granskning av forskningsprojekt, se **Bilaga.**

§ **4** Ordföranden meddelar att nästa sammanträde i avdelning 2 äger rum **onsdagen den 30 mars.**

§ **5** Ordföranden förklarar mötet avslutat.

Christian Groth	Greger Lindberg	Pär Sparén
Ordförande	Vetenskaplig sekreterare	Vetenskaplig sekreterare
